# Peptide Phage Display as a Tool for Drug Discovery: Targeting Membrane Receptors

**DOI:** 10.3390/molecules16010857

**Published:** 2011-01-21

**Authors:** Peter Molek, Borut Strukelj, Tomaz Bratkovic

**Affiliations:** 1 Department of Pharmaceutical Biology, Faculty of Pharmacy, University of Ljubljana, Aškerčeva 7, SI-1000 Ljubljana, Slovenia; 2 Department of Biotechnology, Jozef Stefan Institute, Jamova 39, SI-1000 Ljubljana, Slovenia

**Keywords:** membrane receptors, agonists, antagonists, peptides, phage display

## Abstract

Ligands selected from phage-displayed random peptide libraries tend to be directed to biologically relevant sites on the surface of the target protein. Consequently, peptides derived from library screenings often modulate the target protein’s activity *in vitro* and *in vivo* and can be used as lead compounds in drug design and as alternatives to antibodies for target validation in both genomics and drug discovery. This review discusses the use of phage display to identify membrane receptor modulators with agonistic or antagonistic activities. Because isolating or producing recombinant membrane proteins for use as target molecules in library screening is often impossible, innovative selection strategies such as panning against whole cells or tissues, recombinant receptor ectodomains, or neutralizing antibodies to endogenous binding partners were devised. Prominent examples from a two-decade history of peptide phage display will be presented, focusing on the design of affinity selection experiments, methods for improving the initial hits, and applications of the identified peptides.

## 1. Introduction

Phage display technology is based on the ability to express foreign (poly)peptides as fusions to capsid proteins on the surface of bacteriophage and was first described in 1985 by George P. Smith [1]. Surface display is achieved by inserting a peptide-encoding gene into the gene for a capsid structural protein. Billions of pooled peptides presented on phage particles form a phage-displayed peptide library, and in contrast to regular synthetic small molecule libraries, as many as 10^10^ different peptides can be screened simultaneously for the desired activity [2,3]. Importantly, peptides selected from phage libraries generally target biologically relevant sites on the surface of target proteins (e.g., enzyme active or allosteric sites) and therefore often interfere with the activity of the target protein [4,5]. Over the past two decades, phage display has influenced many scientific fields including (i) drug discovery/design (screening for receptor agonists and antagonists [6,7,8,9,10,11,12], drug target validation [13,14], development of vaccines [15], *in vitro* selection of new antibodies, antibody fragments and antibody surrogates as randomized fragments on diverse scaffold proteins [16,17], discovery of agents for targeted delivery of drugs and gene therapy [18,19]), (ii) proteomics (analysis of protein-protein interactions [20], epitope mapping [21], identification of (novel) enzyme substrates and inhibitors [22,23], improvement of the proteolytic and folding stability of muteins [24]) and (iii) enzymology (designing catalytic antibodies (abzymes) and enzymes with novel specificities [25]). 

Various phage-displayed peptide libraries have been designed using either lytic or filamentous phage or phagemid vectors (thoroughly discussed elsewhere [2,3,26,27,28]). The most common display systems are based on filamentous phages in which peptides are fused to either major (p8) or minor coat proteins (p3). The choice of the coat protein that carries library peptides determines display valency, which can be anywhere between less than one and several thousand copies per virion on average. High-copy display is associated with avidity effects, typically resulting in selection of low-affinity peptide ligands, but can be preferred in specific situations [29,30]. According to Smith’s classification [2], type 8 system stands for p8 phage display where all ~2,700 copies of p8 are transcribed from a single fusion gene on a *phage* vector. If a single *phage* vector carries both recombinant and wild-type g8 genes, this is referred to as a type 88 system. Finally, the p8 *phagemid* display is denoted as an 8 + 8 type system (implying there are two different forms of p8; peptide-p8 fusion-encoding genes are harbored by phagemids, whereas wild-type p8 is contributed by a helper phage). Analogously, p3-display systems are referred to as 3, 33 and 3 + 3, respectively, and typically have significantly lower valencies with a maximum of five copies per virion for the type 3 display. Novagen’s system T7Select for display of peptides and proteins on the capsid of lytic phage T7 also offers the option of adjusting display valency to one’s needs by choosing among phage vectors T7Select-1, -10, and -415 (low, intermediate, and high copy display vectors, respectively) in which major coat protein-peptide fusion genes are transcriptionally controlled by diverse regulatory elements [26].

Expression of short peptides on the phage body is generally well tolerated and can be tailored to encompass a wide range of display valencies. In contrast, proteins, especially large ones, typically disrupt the integrity of the capsid at high copies. Nevertheless, the use of protein scaffolds (e.g. antibody fragments, minibodies, affybodies, knottins, or protease inhibitors; reviewed in [2,17,31,32,33]), in which a part of the sequence dispensable for attaining the correct fold is exchanged for a random stretch of amino acids, is a popular approach for constructing phage display libraries. Locking library peptides to a certain conformation provides the advantage of obtaining high affinity ligands due to lowering of entropic cost upon target binding. However, the scaffold protein needs to be efficiently expressed in the bacterial host and the fusion to capsid structural protein compatible with extrusion across the plasma membrane. Alternatively, peptides can be constrained by cyclization (incorporation of pairs of cysteine residues forming intramolecular disulfide bonds [2]). In this paper we focus entirely on the *peptide* phage libraries as short peptides offer numerous advantages over protein therapeutics (discussed in Section 4).

The majority of pharmaceutical drugs exert their effects by interacting with membrane receptors. Combined with rational drug design, the screening of combinatorial peptide libraries against membrane receptors is a powerful tool for discovering novel pharmacologically active receptor agonists and antagonists or small peptide ligands for the targeted delivery of drugs, genes and diagnostics. Phage display library screening also enables the investigation of ligand-receptor interactions [6,7,8,9,10,11,12,34] because a map of ligand or receptor binding sites can be constructed on the basis of selected peptide sequences [35]. Conveniently, the small size of the selected peptide lends itself to the design of non-peptide mimetics with improved characteristics [36,37]. Here, we review selection strategies for screening phage-displayed random peptide libraries, focusing on the different approaches that have been implemented to make the technology applicable to the selection of membrane receptor ligands. We also discuss how primary screening hits can be optimized for downstream applications.

## 2. General Considerations on Phage Display for Targeting Membrane Receptors

Biopanning is a method for obtaining small numbers of phage clones (each representing an individual peptide) with desired properties (affinity or activity) from an initial bacteriophage pool. The general affinity selection procedure consists of three main steps: (i) introduction of phages to an immobilized target, (ii) removal of unbound phages by washing and (iii) elution of bound phages. Ideally, one cycle of selection should suffice, but in practice, several rounds of selection are necessary (typically two to four) to isolate target-specific binders. Therefore, eluted phages are amplified in host bacteria and subjected to additional rounds of selection, usually under conditions of increased stringency [2]. A negative selection step (*i.e.*, subtractive panning on an otherwise equivalent system that lacks the target) can be introduced before the positive one (panning against the actual target) to further minimize the retention of off-target binders. Following the last selection round, individual clones from an un-amplified eluate are isolated and characterized for target binding and/or biological activity. The primary structure of the displayed peptide is easily identified by determining the nucleotide sequence of the corresponding insert [2,38]. 

Plasma membrane-embedded receptors are indispensable for normal cell-to-cell biochemical and electrical signaling and are involved in all essential physiological functions. Not surprisingly, they comprise the largest group (more than 60%) of drug targets [39,40]. There are numerous types of receptors that vary in their intracellular functions, in the nature of their native binding partners and in the mechanism of ligand-receptor interaction. In general, membrane receptors are composed of three basic parts, the extracellular, transmembrane, and intracellular regions, each of which represent a functionally unique and spatially distinct part of the molecule. For drug development, the most important part of a membrane receptor is its extracellular domain (ectodomain), which contains one or more ligand binding sites. Ectodomains are typically the preferred drug target sites for pragmatic reasons (*i.e.*, accessibility). However, rare compounds, like Tyr-kinase inhibitors, modulate receptor function via an interaction with its intracellular signal-transducing region [41,42]. Peptides are generally too hydrophilic to diffuse across plasma membranes and need to be modified for improved cell penetration. For example, Gubaeva *et al.* [43] identified a receptor-independent peptide modulator of G protein-coupled receptors that interacted with the intracellular Gβγ-subunit, but the peptide was only active *in vivo* after myristoylation. Nevertheless, forced intracellular expression of bioactive peptides represents an alternative to RNAi-based gene silencing for target validation [13,44].

In contrast to soluble proteins, relatively few high-resolution structures of membrane proteins have been determined (*i.e.*, using X-ray crystallography or NMR spectroscopy); in the majority of cases, the three-dimensional folds have been inferred from homology modeling and low-resolution images generated by atomic force microscopy. This hinders rational structure-based design of membrane receptor-targeting drugs [39]. On the other hand, screening phage-displayed libraries for bioactive ligands requires no previous knowledge of the target structure. Usually, the main objective of screening phage peptide libraries against membrane receptors is the identification of novel antagonists, agonists or allosteric modulators (hereafter collectively referred to as receptor modulators). In addition to serving as leads for the further development of clinically applicable therapeutics, bioactive peptides can also be useful in studying the mechanism of a natural ligand-receptor interaction or elucidating the biological role of a particular receptor at the molecular, cellular or *in vivo* level [2,35,38]. Moreover, peptides targeting membrane receptors (especially endothelial ones) can be exploited for the targeted delivery of drugs or diagnostic substances to specific organs or tissues and to mediate cell internalization [2,38,45,46,47,48]. Additionally, high affinity ligands for receptors, through which viruses recognize and enter host cells, have been designed to prevent viral infections [40,49].

Unfortunately, the binding affinities of the isolated peptides are, in general, too low to support their therapeutic use [50]. This is especially true for antagonistic peptides which need to occupy at least half of the receptors to induce an inhibitory effect. On the other hand, these peptides or their further improved versions can be very effective in targeting specific cells or tissues when conjugated with therapeutic or diagnostic agents [51,52,53,54]. Like antibodies, targeting peptides can be attached to the surfaces of different carrier systems such as nanoparticles, liposomes or phage virions [50,54,55,56,57,58,59]. In this context, small peptides have many advantages over antibodies: lower immunogenicity, easier and less expensive production, higher attainable surface density (larger number of peptides per surface unit and consequently higher avidity of such a conjugate), and minor contribution to the increase in particle size and consequently better tissue penetration [36]. One of the most prominent examples of a phage display-derived targeting peptide is the three-amino-acid motif RGD, which targets tumor vascular endothelial cells by binding the αVβ3 integrin, which is exclusively expressed in angiogenic endothelial cells [60,61].

Recent research has illuminated the structure and function of a number of cytokines and growth factors and their idiosyncratic receptors [40]. Cytokines are heterogeneous intercellular signaling proteins that play important roles in the regulation of the immune system, numerous regenerative processes and cancer progression. Cytokines as a group continues to expand and includes interleukins (IL), interferons (IFN), chemokines, and hematopoietic growth factors. Structurally related cytokines or cytokine receptors can be classified into distinct families or classes. Members of the major classes of cytokine receptors (type I and II cytokine receptor families) are all single-membrane spanning membrane proteins that form homo- or heterodi(oligo)meric receptor complexes to activate intracellular signaling. However, it is unclear whether cytokine binding promotes receptor oligomerization or whether pre-formed receptor complexes already exist on the cell surface and subsequent ligand binding solely induces a conformational change, although both events may occur depending on the ligand concentration in the cellular environment [10,62,63,64]. With respect to phage library screening, the nature of cytokine-receptor interactions likely influences affinity selections, and it may be advantageous to immobilize the target receptor for panning at high density to allow the potential ligands to contact two or more receptor molecules simultaneously. Such ligands may consequently trigger appropriate conformational alterations of the receptor complexes, giving rise to intracellular signal transduction *in vivo*. Although the binding of a cytokine to its receptor occurs over a large surface, usually only a few amino acid residues on the ligand and receptor contribute the majority of the binding energy. Alternative small molecule ligands (mimicking the critical binding residues) can potentially be found for any cytokine receptor [6]. Indeed, numerous biologically active peptides identified from phage libraries that act on complex cytokine receptors have been reported [7,8,34,65,66,67,68]. Importantly, even if a peptide does not interact with the ligand binding site on the receptor, it can still possess biological activity. For example, the peptide may modulate receptor activity allosterically and influence either native ligand binding or subsequent signal transduction. Also, peptides that bind selectively to a monomeric receptor molecule might prevent receptor oligomerization and act as antagonists *in vivo* without interfering with ligand binding [10,62]. In some cases, an antagonistic effect can be achieved by merely altering the orientation of the individual receptor monomers even though they are still associated in a complex [69]. These alternative antagonizing mechanisms highlight the importance of thoroughly examining all ligands because the inability of a peptide to block the ligand-receptor interaction *in vitro* does not necessarily indicate a lack of biological activity *in vivo*. However, the opposite may also be true: many peptides that are active *in vitro* will not retain activity when applied *in vivo* as a result of low bioavailability (inaccessibility to the site of action) and high clearance rate (fast elimination from the body; see Section 4.2). 

Compared to cytokine and growth factor receptors, far less success has been achieved in screening phage-displayed peptide libraries against G protein-coupled receptors (GPCRs) (reviewed in [5]), which constitute the largest class of drug targets [70]. The main restriction limiting the use of GPCRs as targets in biopannings is the complex structure of their extracellular ligand binding region. This region is comprised of several distinct parts, including the *N*-terminal chain, parts of the seven transmembrane helices, and three connecting loops. Its structure thus strongly depends on maintaining the integrity of the whole molecule, which is buried in the cell membrane.

## 3. Biopanning Strategies on Membrane Receptors: From Single Molecules to Organisms

One method frequently used to identify peptide receptor modulators is to screen a library consisting of continuous fragments of the natural receptor ligand. Any part of the ligand that binds but does not activate a particular receptor would be a suitable candidate for antagonist design. However, the chances of successful selection increase when using a library of random peptides because peptide ligands that are non-homologous to the primary structure of the natural ligand can be identified [71]. Such peptides are called mimotopes because they mimic assembled, discontinuous “epitopes” of the natural ligand (composed of amino acids at distal positions within a primary sequence that become proximal in the properly folded protein) [72]. Although the identification of receptor agonists using the same approach seems intuitively unlikely, some peptides have been shown to possess intrinsic activity and trigger receptor activation [6,7,65,73,74]. 

**Figure 1:**
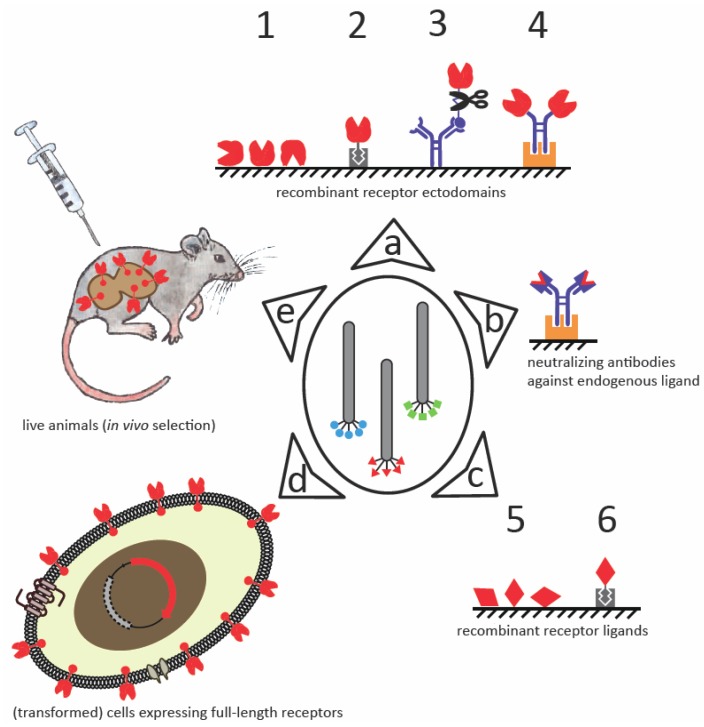
Schematic representation of some well-established biopanning approaches to identify membrane receptor-binding peptides from phage-displayed libraries. **(a)** Due to the difficulties accompanying isolation and recombinant expression of whole membrane receptors, only the extracellular regions (shown in red) or their individual domains can be used as target molecules. These can be immobilized to an appropriate matrix (oblique striped grounding) by direct adsorption (1) or by a suitable affinity tag that is recognized by its respective binding partner (e.g., biotin-avidin interaction, 2) or a specific antibody (3). A linker connecting the affinity tag to the receptor fragment that contains a specific protease cleavage site (3) can be introduced to facilitate the specific elution of target-bound phages by protease treatment. Alternatively, receptors may be imitated by recombinant antibody chimeras (receptor ectodomains fused to the Fc-region of an antibody; 4), in which case the target molecule is specifically captured to protein A- or G-coated matrix (shown in orange). **(b)** Targeting neutralizing antibodies against the endogenous ligand can result in selection of peptides binding to cognate receptor. **(c)** The ligand-receptor interaction can be inhibited by targeting the endogenous ligand instead of the receptor. Again, direct (5) or indirect immobilization (6) of the ligand is possible. **(d)** A more advanced strategy is to screen against whole cells over-expressing the full-length membrane-embedded receptor; this is usually achieved by transforming cells with a gene encoding the receptor. **(e)** In the *in vivo* approach, library phages are injected intravenously into an animal, and specific binders are recovered from tissue biopsies. The identities of the targeted receptors are determined afterwards.

The use of isolated whole membrane receptors as targets in the *in vitro* setting of library screening is problematic because membrane receptors are extremely hydrophobic and/or maintain their native fold only in a lipid bilayer environment [39,75,76]. Interestingly, a recent report [77] demonstrated that at least some membrane proteins can be expressed in a correctly folded full-length form when tethered to filamentous phage. The large phage body reportedly improves solubility so that the high hydrophobicity of the protein is no longer an issue. Theoretically, filamentous phage-presented whole receptors could serve as targets for affinity selection of peptides from libraries displayed on a different type of phage, e.g., T7, or *vice versa* (analogous to the screening of an antibody fragment library against a phage-displayed peptide target reported by Castillo *et al.* [78]). However, alternative targets to full-length receptors are typically used for biopanning (Figure 1). These include smaller recombinant fragments (e.g., soluble ectodomains) or their chimeric fusions with other proteins [8,9,74,79] and neutralizing antibodies to endogenous binding partners [65,68,80,81]. In addition, panning against an endogenous receptor ligand can be another strategy for obtaining peptides that block the ligand-receptor interaction [82,83,84,85]. For example, Fairbrother *et al.* [85] identified indirect peptide antagonists of the vascular endothelial growth factor receptor (VEGFR) by targeting the receptor-binding domain of VEGF. If panning against full-length membrane-embedded targets is desired, whole cells can also be used to display (recombinant) receptors [46,49,86,87]. Finally, *in vivo* selection can be performed to obtain peptide ligands of membrane-embedded receptors, especially if they are over-expressed in a tissue-dependent manner [19,66,73]. The *in vivo* selection approach is especially attractive when searching for new moieties for targeting specific cells. In this context, identification of the exact target structure on the cell surface is of secondary importance. Nevertheless, the identity of the targeted receptor can sometimes be subsequently inferred from the peptide binders if they mimic receptor binding fragments of the endogenous ligand [47,73,86,88]. A selection of membrane receptor-targeting peptides that have been identified by screening phage-displayed libraries is given in Table 1.

### 3.1. Recombinant Receptor Mimetics

Several different soluble receptor mimetics (variants of ectodomains) have been successfully used to select peptide modulators of membrane receptors. These mimetics were either covalently bound or adsorbed to a solid support or immobilized to an appropriate matrix indirectly (e.g., via chemically attached groups (biotin) or a fusion affinity tag) [2,37,95,96]. A popular strategy for large extracellular receptor regions (especially homodimeric) is fusion to the Fc-region of immunoglobulins (IgG) [37,97,98] and subsequent capture on a protein G- or A-covered matrix.

Indirect target attachment offers some important advantages: (i) uniform target orientation with a markedly reduced probability of denaturation (which may occur by non-specific adsorption) [38], (ii) minimal target consumption (due to the highly specific and strong interactions between the labeled target and the capturing molecules), and (iii) the potential to allow the phages to interact with the target in solution and subsequently capture on a solid phase the phage-target complexes (known as the “solution binding” approach) [2,38]. With solution binding, undesired avidity effects (caused by multivalent peptide display) are minimized, and the stringency of the selection can be easily controlled by adjusting the target concentration [2,99,100,101]. Matrices other than microtiter plates, such as beads [37,47,51,102,103] or chromatographic columns [38,93,104], can also be used for both direct or indirect attachment of target molecules. Chromatographic affinity selection allows the identification of high affinity binders in a single panning cycle, which reduces the overall duration of the experiment and avoids the introduction of amplification-dependent bias [104,105].

#### 3.1.1. Recombinant Receptor Fragments

In most studies, soluble ectodomains have been used to isolate bioactive peptides that target membrane receptors. For example, Vrielink *et al.* [37] screened a library of disulfide-constrained random peptides to identify death receptor 5 (DR5) antagonists. DR5, activated by TRAIL (TNF-related apoptosis-inducing ligand), is the main receptor for mediating the signal for apoptosis [40,71], and antagonizing its activity might be beneficial for the treatment of neurodegenerative and autoimmune diseases. Affinity selection was carried out against the DR5 ectodomain-IgG Fc-fusion captured on protein A-coated paramagnetic beads. To direct selection against the receptor ectodomain, phages were subjected to negative screening against the Fc-region. Moreover, phages were eluted with the recombinant TRAIL to further enrich for clones targeting the ligand interaction site on DR5. Synthetic peptides identical to those isolated by phage-display, as well as peptide dimers generated in parallel and antiparallel orientations due to nonspecific double intermolecular disulfide bond formation, bound Jurkat cells expressing DR5 in a concentration-dependent manner and reduced TRAIL-induced cell death in Colo205 colon carcinoma cells [37].

Membrane receptors usually have large multidomain extracellular regions, which can potentially interact with diverse peptide sequences. The isolated ligand-binding domains of receptors are excellent surrogate targets for the focused selection of peptides that mimic endogenous ligands. Targeting shorter receptor fragments may also improve specificity for a particular member of a highly conserved receptor family. On the other hand, a synthetic peptide that corresponds to a particular receptor domain may adopt a different conformation than in the native protein, so the selected peptide may not recognize the receptor in its natural environment [38]. Therefore, it may be necessary to first screen a phage library against a synthetic peptide and then subject the resulting enriched library to affinity selection for binding the native protein (see Section 3.4) [38,93].

Some heteromeric receptors share a common membrane-spanning subunit, typically devoted to signal transduction, while other unique subunits interact with specific cytokines to trigger signaling. To identify selective receptor modulators, the common subunit should be avoided as a target. With that in mind, Su *et al.* [8] depleted a library of phages that bound the gp130 subunit, a common signaling co-receptor for IL-6 and several other cytokine receptors, before exposing the library to the IL-6Rα subunit, thereby recovering ligands selective for IL-6R. One peptide suppressed IL-6-induced tumor growth in an animal model, suggesting cancer therapy potential. In another report, Hetian *et al.* [9] identified peptide modulators selective for VEGFR2, also known as the kinase insert domain-containing receptor (KDR). VEGF is one of the most important factors in both physiological and pathological angiogenesis and activates its cognate receptors VEGFR1-3. KDR is a tyrosine kinase receptor which, after VEGF-induced dimerization, mediates the majority of the biological activities of VEGF related to proliferation, differentiation, and migration of endothelial cells [106,107]. A random dodecapeptide library was screened against the fusion protein GST-KDR (extracellular domains I-IV of KDR fused to glutathione S-transferase) after being subjected to negative selection against three additional GST-fusion proteins (including VEGFR1). One of the recovered peptides competed with VEGF for binding to KDR and exerted anti-angiogenic activity *in vitro* (*i.e.*, prevented human endothelial cell proliferation) and *in vivo* (*i.e.*, inhibited angiogenesis in the chick embryo choroallantoic membrane and reduced tumor growth in mice) [9].

Two of the earliest and probably most prominent reports of the identification of potent *agonistic* peptides by phage library screening were contributed by Wrighton *et al.* [6] and Cwirla *et al.* [7] for the erythropoietin receptor (EpoR) and thrombopoietin receptor (TpoR), respectively. EpoR and TpoR are both members of a large hematopoietic cytokine receptor superfamily, which includes receptors for most interleukins and various other growth factors. Notably, peptides selected for binding EpoR and TpoR share a similar amino acid motif (GPLT and GPTL, respectively) and form a β-turn conformation in solution. Dimerization of EpoR itself is not sufficient for intracellular signaling [69,108], and it appears that the Epo mimetic peptide and, by extrapolation, Epo itself cause conformational changes in the receptor ectodomains that bring the cytoplasmic domains of the receptor subunits together and finally lead to intracellular signal transduction [63]. Wrighton *et al.* [6] used the soluble ectodomains of EpoR fused to the *C*-terminal sequence of the human placental alkaline phosphatase (HPAP) to capture the target protein on a microtiter plate via immobilized anti-HPAP monoclonal antibody (mAb). The specific exposure of EpoR ectodomains bound to a bivalent antibody may have favored the selection of peptides that interacted with the EpoR dimer like an agonist, as proposed by Dower [109]. Bound phages were released by cleaving the linker connecting EpoR to HPAP with thrombin, whereas unspecific elution using an acidic buffer did not enrich for specific EpoR binders. The initially selected binding clone was used as a template for the construction of partially degenerated low-valency phagemid libraries containing peptides of increased length. During the additional screenings, an increasing concentration of Epo was added to the selection media to compete away phages weakly bound to the receptor. The newly selected erythropoietin mimetic peptides (EMPs) specifically stimulated erythropoiesis *in vivo* despite an apparent lack of similarity to the erythropoietin primary structure. It was later shown that the EMPs form homodimers via hydrophobic interactions to promote both the dimerization and activation of EpoR [110]. Intrinsic activity of a representative peptide agonist, EMP1, was improved by covalently joining two molecules of EMP1 via a short linker. The EMP1 sequence was minimized without a significant loss of activity [111], and the peptide dimer was PEGylated to give rise to peginesatide (Hematide, Affymax) [112], which is currently in phase III clinical studies for the treatment of anemia [113,114,115,116]. 

The selection strategy that led to the identification of TpoR peptide agonists was similar to the one described above for EpoR activators. In addition to phage-displayed libraries, Cwirla *et al.* [7] screened libraries of random peptides fused to the *Escherichia coli* lac repressor (LacI) protein (*i.e.*, peptides-on-plasmids libraries) against TpoR. The initial selection yielded two distinct families of small peptides with affinity to TpoR. As with EpoR, the sequences of the TpoR-binding peptides were not homologous to the primary structure of the endogenous ligand. Several non-phage mutagenesis libraries were constructed and used in affinity maturation of the TpoR ligands. A covalently linked dimeric form of a 14-amino acid peptide (AF13948) was as potent as the natural thrombopoietin in cell-based assays. On the basis of AF13948, Amgen developed a peptibody termed romiplostim. Each of the two identical subunits of the peptibody consists of a Fc-region of human IgG1 fused to two consecutive TpoR-binding peptides [117]. Romiplostim (marketed under the trade name Nplate) was approved in 2008 by the FDA for the treatment of idiopathic (immune) thrombocytopenic purpura [118]. Both EpoR and TpoR peptide agonists were selected solely based on their ability to bind to the corresponding receptor, which demonstrates that the biological activity of a peptide ligand (*i.e.*, whether it acts as an agonist or an antagonist) cannot be predicted in advance. The assumption that ligands recovered from peptide libraries will simply displace the endogenous ligand from the receptor, thereby blocking signal transduction, is thus not necessarily valid.

Karasseva *et al.* [79] identified a peptide, p6.1, that bound to human ErbB-2 tyrosine kinase receptor with a relatively modest affinity. ErbB-2 (also known as HER2) is a member of the epidermal growth factor (EGF) receptor family and is implicated in many human malignancies. Although ErbB-2 itself cannot apparently bind any ligand from the EGF family, it acts as a preferred partner in heterodimeric complexes with other ligand-bound ErbBs to transduce signaling. Selection from a phage library was carried out against a biotinylated ErbB-2 ectodomain pre-captured on streptavidin-coated microtiter plates. The amount of target was gradually reduced to increase the stringency of the selection. Because p6.1 binds tumor cells bearing ErbB-2, it has potential as a tumor imaging agent or a vehicle for the specific delivery of radionuclide or cytotoxic agents to tumors overexpressing ErbB-2 [89].

#### 3.1.2. Neutralizing Antibodies against Endogenous Binding Partners

Extracellular portions of integral membrane receptors, such as GPCRs, cannot be expressed as soluble recombinant proteins to be used as targets in affinity selections. The conformation of a GPCR’s ligand-binding site depends critically on the integration of the whole receptor molecule in the lipid bilayer. Neutralizing antibodies against endogenous ligands can, however, serve as alternative targets for the identification of bioactive peptides. Here, the assumption is that the antibody’s antigen-binding regions mimic the receptor’s ligand binding site, thereby providing a “mold” for the recovery of peptides that will cross-react with the receptor. Bonetto *et al.* [81] have successfully identified melanocortin receptor 1 agonistic and antagonistic peptides by screening phage libraries against three monoclonal anti-adrenocorticotropic hormone antibodies. Peptides that target growth factor [80] and cytokine receptors [65,67], which were selected by binding the corresponding antibodies, have also been reported. In a number of cases, the use of neutralizing antibodies as targets has proved to be a superior strategy to directly screening against receptors or their structural parts [65,67,68,80]. This might be, in part, because the bivalent nature of antibodies contributes to a higher avidity of the phage-target interaction. Moreover, antibody CDRs are generally more surface-exposed than receptor binding sites because of the physiological roles of both types of molecules. Antibody structure is optimized in a way that enables rapid and effective antigen binding, often at the expense of specificity [119,120], which is supported by the widespread appearance of antibody cross-reactivity. On the other hand, receptors must distinguish between highly structurally related ligands from the same family, although this may be accompanied by individual binding sites with relatively low affinities. This is especially true for cytokine receptors with large and often multimeric binding sites. A much higher number of hot spot residues than is found in antibody CDRs ensures specific binding of the cytokine while preventing strong interactions with short peptides. At the same time, receptor interaction sites can be sufficiently buried in the ectodomain to render them inaccessible to peptides expressed on a large phage body while still possibly accommodating free peptides.

Neutralizing antibodies, however, do not necessarily recognize the exact receptor-binding epitopes located on ligands but may exert their antagonistic effect by sterically blocking the ligand-receptor interaction by binding to sites proximal to these regions. Indeed, we have observed that a cyclic peptide selected against a monoclonal human leptin-neutralizing antibody competed with recombinant leptin for binding to the antibody but had no affinity for the recombinant leptin receptor ectodomain (unpublished data). We later noted that the peptide has some sequence similarity to one of the loops that connects helices known to constitute the receptor binding site.

### 3.2. Panning against Whole Cells

Phage libraries can also be screened against living cells expressing the membrane receptor of interest. This eliminates concerns about misfolding of the target protein and allows for selection against the extracellular regions of receptors in their native conformations. The targeted cells can either be in suspension or attached to a culture dish.

The majority of reported pannings against whole cells employed different cancer cells [45,46,47,51,86,87,94]; the main goal was to find peptides that bound tumor-specific receptors that could be used for the targeted delivery of drugs or diagnostic agents. Depending on the type of cell culture, selection procedures can be classified as *ex vivo* or *in vitro*. Hoffman *et al.* [121] used the term *ex vivo* phage display to denote screenings against primary cell suspensions of organs and tissues that contain different types of cells (e.g., endothelial and tissue parenchymal cells). In contrast, the term *in vitro* phage display describes selections against immortalized cell lines (*i.e.*, homogeneous populations of transformed cells). Alternatively, a cell line can be stably transfected to express the receptor of interest at high copy numbers [49,121]. The composition of the plasma membrane is extremely complex, with diverse protein and carbohydrate structures that act as potential decoys in affinity selections from phage libraries. Therefore, high cell surface density of the membrane receptors representing the actual targets favors the enrichment of relevant peptides. Additionally, subtractive panning against untransfected cells (thus lacking the target receptor) is strongly recommended to limit the recovery of target-unrelated peptides. In selections performed *ex vivo*, subtraction is carried out by incubating the library with similar but not identical cells (e.g., normal instead of tumor cells) [45,87,94].

In one example of an *in vitro* selection, Wang *et al.* [49] transfected Chinese hamster ovary (CHO) cells with vector harboring the human chemokine receptor 5 (CCR5) gene. CCR5 belongs to the G protein-coupled receptor (GPCR) family of integral membrane proteins. It is involved in a range of human diseases (e.g., multiple sclerosis, rheumatoid arthritis, and renal allograft rejection) and mediates HIV-1 cell entry in concert with CD4 [122,123]. A library of phage-displayed random dodecapeptides was reacted with the transfected cell line in suspension, and the phages that bound to cells were separated from the unbound subpopulation via multiple rounds of centrifugation and washing. Two consecutive subtractive pannings against the untransfected CHO cells were performed before each positive selection step. After four rounds of selection, a peptide was identified that specifically bound CCR5 and inhibited the biological activity of RANTES (an endogenous ligand of CCR5). In another study [12], HEK 293 cells were transfected with the cDNA for glycine receptor α1 (GlyRα1) and used as “target bearing vehicles” to screen for peptide modulators of GlyRα1. The glycine receptor is the major mediator of inhibitory neurotransmission in the spinal cord and brainstem and belongs to the Cys-loop superfamily of ligand-gated ion channels [124]. Untransfected cells were used in the negative selection steps. Phage display technology was combined with electrophysiological testing to identify peptides that modulated GlyR function without affecting the closely-related GABA receptors. Frog oocytes were transformed with either the glycine or the GABA receptor cDNAs, and the transmembrane ion current was measured after treatment with glycine and GABA alone or in combination with individual synthetic peptides. None of the reported peptides had any effect on the transmembrane ion current in the absence of glycine. However, some peptides selectively attenuated while others enhanced the effects of glycine and thus, most likely acted as negative and positive allosteric modulators of GlyRα1, respectively [12].

Giordano *et al.* [35] developed an innovative method for separating cell-bound phages from free phages in solution during the affinity selection. Termed BRASIL (biopanning and rapid analysis of selective interactive ligands), the method is based on differential centrifugation of an aqueous suspension containing mixed phages and cells through a lower non-miscible organic phase. Free virions are retained in the upper aqueous phase, while the phages that are anchored to cells are pelleted in the organic phase. Typically, a single centrifugation step is sufficient for selective recovery of cell-bound phages. Giordano *et al.* [35] used the technique to screen for cyclic peptides that interacted with VEGFR1 and neuropilin-1. Neuropilin-1 (NRP1) is a membrane-bound co-receptor of different tyrosine kinase receptor families [125]. Before being exposed to VEGF-stimulated human umbilical vein endothelial cells (HUVECs), the phage library was first depleted of unspecific binders by exposure to HUVECs grown in the absence of VEGF (VEGF stimulation increases the expression of VEGFR). Interestingly, a peptide with the amino acid sequence CPQPRPLC, which was identified after three selection rounds, was a dual ligand that interacted with both VEGFR1 and NRP1 [35]. Only a portion of this peptide, the tripeptide RPL, was later shown to be responsible for binding both receptors [90]. Because of its small size, RPL is a perfect peptide lead for the design of a novel class of small VEGFR inhibitors.

When screening phage libraries against whole cells, it is worthwhile to lyse the cells and recover phages that might have translocated into the cell interior by receptor-mediated endocytosis [45,46,51], especially if the goal is to identify peptides for intracellular delivery of therapeutic or diagnostic agents. If the selective recovery of only internalized phages is desired, extracellular phages can be inactivated by the protease subtilisin. Chloroquine can be added to cells during panning to increase membrane stability and inhibit degradation of internalized phages [46]. Zhu *et al.* [45] screened a peptide library against human hepatocellular carcinoma (hHCC) cells and found that only internalized phages were enriched for peptides with a highly conserved motif, whereas the surface-bound phages (recovered by elution with an acidic buffer) did not display homologous peptides.

### 3.3. In Vivo Selection 

For *in vivo* selections, library phages are injected intravenously into laboratory animals. A notable difference between *in vivo* and *ex vivo* approaches is that phages are primarily exposed to the vascular endothelial cells in the *in vivo* method but are exposed to all cell types from a particular tissue in the *ex vivo* variation [121]. Compared with *in vivo* phage display technology, cell-based panning is simpler and more effective. Capillary vessels of the vascular system may act as impenetrable barriers for phage passage, so the majority of phages recovered by *in vivo* panning actually bind to the vascular endothelium cells and not tissue parenchymal cells. Hence, it is not surprising that the use of *in vivo* phage display technology has led mainly to reports of specific vascular endothelium cell-binding peptides [19,60,126]. Considering the size of vascular fenestrations [127], *in vivo* targeting of a bacteriophage to tumor cells is theoretically possible (at least with smaller lytic phages) but would require a longer circulation time than targeting the vascular endothelium [47]. Hoffman *et al.* [121] have developed *in vivo* selection schemes using phage-displayed libraries to discover peptide and protein ligands for macromolecules that are expressed in an organ- and tissue-specific manner. Following injection of the phage library, phages are rescued from different organs or tissues and can be amplified and purified for potential iterations of selection. Vascular perfusion can be used to remove unbound phages, and additional *ex vivo* washing of tissue-isolated cells is also favorable. When looking for peptides that target *human* cancers, tumors can be implanted into laboratory mice in the form of xenografts, and the animals can be subjected to *in vivo* phage display [128].

A major breakthrough in *in vivo* selection was achieved by Arap *et al.* [19], who identified numerous peptides that targeted the human vasculature. They administered a random disulfide-constrained heptapeptide phage library to a cancer patient, and tissue biopsies were obtained 15 minutes after infusion to recover phages from various organs. Due to ethical concerns, only one round of selection was performed, which required a large input of phage library and the determination of a huge number of peptide sequences. Overall, 47,160 phage inserts that were recovered from the representative samples of five tissues and from the unselected library were analyzed. Computational processing of the amino acid sequences identified several peptide motifs that targeted organ-specific vasculatures. One of the peptides, CGRRAGGSC, was later shown to specifically bind the α subunit of the interleukin 11 receptor (IL-11Rα) [73]. As a member of the hematopoietic cytokine receptor family, the IL-11Rα chain forms an active receptor complex with the gp130 transducing subunit upon IL-11 binding [10]. IL-11 was initially characterized as a cytokine with thrombopoietic activity and was later found to have pleiotropic effects in multiple tissues. Notably, the peptide targeting IL-11Rα bears significant similarity to a region in the native IL-11 (RRAGGS; residues 112-117) that was not previously believed to be involved in receptor binding. The peptide induced cell proliferation through IL-11Rα-mediated STAT3 activation, which was reduced upon addition of soluble IL-11Rα [73]. This observation indicated that the peptide acted as a specific IL-11Rα agonist and may, in this context, represent a novel peptide lead for the design of drugs to prevent chemotherapy-induced thrombocytopenia. Moreover, the peptide CGRRAGGSC enables the targeting of IL-11Rα-expressing cells and subsequent cellular internalization of conjugated small oligopeptides or larger particles (e.g., phages expressing the IL-11 mimic peptide). Because of the increased stage-specific expression of IL-11Rα on prostate cancer cells and tumor endothelium during disease progression, this peptide could serve as a ligand for the targeted delivery of drugs and imaging agents in patients with prostate cancer [66].

### 3.4. Combining Selection Strategies

Different strategies can be combined to improve the efficiency of affinity selection. For example, in a search for IFN-α2b antagonists, Tian *et al.* [67] initially panned a phage-displayed peptide library against immobilized human amnion WISH cells expressing interferon-α/β receptor (IFNAR) four times (eluting with recombinant IFN-α2b) before exposing it to polyclonal anti-IFN-α2b antibodies for three additional rounds of selection. Three groups of structurally related peptides with similarities to IFN functional domain amino acid sequences were recovered. Two peptides were synthesized and shown to interfere with IFN-α2b protection of WISH cells against vesicular stomatitis virus-induced cytopathic effects. 

Grover and co-workers [38,91,92,93,129] have developed an even more complex screening approach while searching for modulators of plasma membrane Ca^2+^ ATPases (PMCAs, also known as Ca^2+^-Mg^2+^-ATPases). PMCAs are ubiquitous transmembrane proteins that function in the regulation of intracellular calcium levels. Four different isoforms exist, and they are expressed in a tissue-dependent manner [91]. Identification of a PMCA4-specific allosteric inhibitor (termed caloxin 1c2) required four separate selection phases performed with two different matrices for target immobilization: a microtiter plate and a calmodulin-sepharose chromatographic column. In phase 1, three rounds of biopanning using a microtiter plate coated with synthetic PCMA4-extracellular domain peptide conjugated to keyhole limpet hemocyanin were performed. Chromatographic columns with calmodulin-sepharose as a stationary phase for the immobilization of PMCA purified from erythrocyte membranes were used for the subsequent phases of selection. Because PMCA binds calmodulin only in the presence of Ca^2+^, the PMCA-bound phages were eluted simply by omitting Ca^2+^ from the buffer. Two selection rounds were carried out in phase 2; negative selection steps against calmodulin-sepharose preceded the positive ones with immobilized PMCA. In phase 3, a large number of phage clones from the final eluate of phase 2 was sequenced, and individual clones were pooled in equal ratios. The resulting library was screened in the same manner as in phase 2, exploiting the competition for target binding between individual phage clones to identify caloxin 1b1, which had a higher affinity for PMCA4. Finally, in phase 4, the authors created a focused library by limited mutagenesis (see Section 4.1) of caloxin 1b1 from which caloxin 1c2, which possessed improved activity and selectivity for the PMCA4 isoform, was selected [38].

## 4. Further Optimization of Selected Peptides

Peptides typically suffer from low oral bioavailabilities and short biological half-lives, making them poor drug candidates. On the other hand, short peptides generally show high selectivity and specificity for their targets and have low systemic toxicity. Numerous structural modifications can be introduced into peptides to further broaden the chemical space and develop analogues with improved pharmacokinetic and/or pharmacodynamic characteristics [130,131]. 

Short peptides that bind to target receptors or cognate ligands with reasonable affinity and specificity can be considered alternatives to antagonistic antibodies. There are several advantages that therapeutic peptides have compared to antibodies: (i) lower manufacturing costs due to synthetic production, (ii) higher activity per mass, (iii) lower royalty stack due to a simpler intellectual property landscape during discovery and manufacturing, (iv) greater stability (longer shelf-life), (vi) generally lower immunogenicity, and (vii) better organ or tumor penetration [36,132]. Below, we briefly review some common approaches for improving peptides selected from phage-display libraries to allow their application as drugs or diagnostic agents.

### 4.1. Optimization of Amino Acid Sequence (Affinity Optimization)

Biological peptide libraries are typically much larger than their synthetic counterparts, which can strongly affect the outcome of affinity selection. The higher the diversity (*i.e.*, the repertoire of phage-displayed polypeptides), the higher the success rate of attaining high affinity ligands. Nevertheless, the strongest binding peptide may be underrepresented or even missing in the initial library (this is especially true for libraries of longer peptides) or may not be efficiently recovered during elution steps, leading to preferential enrichment of weaker binders. To obtain truly high-affinity peptides, a second generation library (focused library) can be constructed on the basis of the primarily selected sequences. Most often, affinity maturation libraries are constructed using either soft or hard randomization of the DNA sequence encoding the peptide with the highest binding affinity or that is most frequently represented. In *soft randomization*, each residue is mutated to a limited extent, and the overall sequence remains similar to that of the parent peptide. The theoretical frequency of amino acid substitution at any position depends on the quantitative ratios between the nucleotides added at particular steps during the synthesis of the oligonucleotides encoding the displayed peptides [133,134]. For example, Fairbrother *et al.* [85] synthesized mutagenesis inserts in a 80:7:7:7 manner, indicating that a mixture of 80% original base and ~7% each of the other three bases was added in each step, resulting in an amino acid mutation frequency of about 40% at each position. Conversely, in *hard randomization* degenerate codons encoding all twenty natural amino acids are introduced at defined positions of the peptide-encoding oligonucleotide. This can facilitate the identification of optimal amino acid residues in weakly conserved motifs [74,83] and the extension of primarily isolated peptides [6]. However, a “milder” randomization approach (called *tailored randomization*) is often used to substitute a particular parental residue (or set of residues) with an amino acid from a limited subset of natural amino acids that may share a common characteristic (e.g., hydrophobic or polar residues, *etc.*). Several amino acid subsets, determined by partially degenerate codons, can be introduced at each position. The derived consensus may differ from the original one depending upon which residues are allowed to co-vary within a library. This is most likely due to cooperative intramolecular interactions between peptide side chains [74].

All of the mutagenesis methods discussed above may be combined to construct a single focused library, but a more rational approach is to begin with soft randomization and later construct a tertiary library by site-directed hard randomization [85,134]. It may also be useful to use a polyvalent library initially and later switch to monovalent display vectors for the construction of mutagenesis libraries to eliminate avidity effects and identify peptides with high intrinsic affinities [6,74,83,85,100].

Ultimately, peptides must be chemically synthesized or expressed using recombinant DNA technology to be amenable to thorough evaluation. The alanine scanning method, in which individual amino acid residues are systematically replaced by alanine, can be used to assess the contribution of each residue to binding affinity, specificity and/or activity. The minimal effective portion of a ligand can be further determined by gradually truncating the peptide’s terminal ends [111].

### 4.2. Options for Improving the In Vivo Fate of Peptide Drug Candidates 

The main disadvantage of peptide drugs is their short half-life *in vivo* (on the order of minutes) due to rapid degradation by proteases, extensive renal filtration, and uptake to other tissues [112,132]. Consequently, the biological activity of a peptide directly depends on its stability in serum. One method to improve pharmacokinetics is to incorporate unusual amino acids, such as *D*-enantiomeric amino acids, in synthetic peptides [36,131]. An elegant way to obtain peptide ligands in the *D*-conformation was described by Schumacher *et al.* [135] and termed mirror image phage display. In principle, the selection is carried out against the mirror image of the original target molecule using a phage library of peptides in the naturally occurring *L*-conformation. The targeted polypeptide has the same amino acid sequence as its natural counterpart but is fully composed of *D*-enantiomeric amino acids. The ultimately selected peptide of interest is translated into its *D*-enantiomeric form, which should interact with the original target protein composed of natural *L*-amino acids. However, the required synthetic preparation of target molecules in the *D*-conformation is a serious obstacle because larger proteins are difficult to synthesize. Nevertheless, mirror image phage display should be considered a worthwhile and viable option due to the considerable advantages of *D*-peptides over *L*-enantiomers. Most importantly, they are resistant to gut and serum proteases, a feature that can dramatically increase their serum half-life [131]. *D*-isomer peptides are also less immunogenic [136] due to the inability of antigen-presenting cells to process such peptides.

The pharmacokinetic properties of peptides can also be improved by synthesizing retro-inverted (retro-inverso) analogues of bioactive *L*-peptides. Here, *retro* refers to the reversed direction of the primary sequence, and *inverso* denotes the alteration in chirality of each individual residue to the *D*-enantiomer. Retro-inverted peptides retain a strong topological correlation to the parent peptide because the side-chain disposition is similar in both (the positions of side-chains are preserved). Thus, the binding affinities of retro-inverted peptides for their cognate targets usually remain unchanged relative to the parent *L*-peptide [82,131]. 

The biological activity of short peptides is commonly potentiated by simply joining monomeric peptides into di-/tri- or higher oligomeric forms. For instance, the potency of both EpoR and TpoR peptide agonists was markedly improved by the formation of their respective dimeric forms; the effect was not due to increased avidity of the conjugates but because the active forms of the two agonists are actually dimeric peptides that must pair non-covalently in the absence of covalent fusion [6,7,110,137]. Different linkers are used to conjugate monomeric peptides to one another, such as small flexible molecules (e.g., a few ethylene glycol units) or larger carriers (e.g., a leucine zipper variant or the IgG Fc-region) [74]. In addition, ligands with non-overlapping sites on the receptor can be coupled to form *peptide concatemers*. The dissociation constant (K_d_) of such a heterooligomer-target complex is usually much lower than the K_d_ of the target and either component alone [38] due to the increased interaction area [63]. 

An important consideration with peptide drugs is their short residence time in the body. To overcome weaknesses related to rapid elimination, peptides are either PEGylated (coupled to a polyethylene glycol chain, e.g., peginesatide [138]), directly fused to a larger protein molecule (such as the IgG Fc-region, e.g., romiplostim [118]), or attached to a group that adheres to serum albumin [139,140]. In general, decreasing immunogenicity, enhancing protease resistance, limiting renal filtration, and actively recycling IgG- and albumin-peptide fusions by the neonatal Fc receptor (FcRn) all contribute to a prolonged biological half-life [138,141].

## 5. Future Prospects

Two of the most advanced drugs developed by screening phage-displayed libraries of random peptides are the PEGylated erythropoietin receptor agonist peginesatide (Hematide, Affymax) and the Fc-fused thrombopoietin receptor agonist romiplostim (Nplate, Amgen). Two other drugs, adalimumab, an anti-TNF-α antibody [142], and ecallantide, a small-protein inhibitor of the plasma protease kallikrein [143], have been developed using phage display, putting this technology side-by-side with other well-established approaches in the field of drug discovery.

Recent exciting advances in the technique predict a bright future for phage display therapeutics. For example, Tian *et al.* [144] and Sandman *et al.* [145] have incorporated genetically-encoded non-natural amino acids into phage-displayed peptides, thereby paving the way for combinatorial libraries of even wider chemical diversities (potentially applicable to the construction of second generation affinity maturation libraries). Furthermore, Woiwode *et al.* [146] designed a hybrid system of phage display and synthetic chemistry by chemically coupling synthetic compounds to the bacteriophage capsid on a one-compound-one-clone principle (each compound was encoded by a unique nucleotide sequence inserted in a non-coding region of the phage genome). Screening a library of folic acid analogues against KB carcinoma cells over-expressing folate receptors resulted in the identification of several folic acid mimetics. Additional approaches are being developed to better control screening conditions [147,148]. Highly sensitive biosensors, such as Biacore (based on surface plasmon resonance) and quartz crystal microbalance apparatuses (based on the piezoelectric effect), not only enable screening against minute amounts of target but, more importantly, also allow the monitoring of phage-target interactions in real time. This makes it possible to empirically modify the stringency of the washing and elution steps during a single experiment and recover truly high affinity binders. Considering the emergence of novel library designs and innovative selection strategies, we are confident that phage display will continue to provide a wealth of peptide leads to be used for the development of new membrane receptor modulators and agents for the targeted delivery of therapeutics and diagnostics. 

## Figures and Tables

**Table 1:** Selected reports of peptides identified from phage-displayed libraries that home to membrane-embedded proteins. If the exact nature of targeted protein on primary cell cultures or tissue xenografts was not determined, it is listed as “unknown” followed by cell culture or tissue type in square brackets. N/A, not available.

Targeted protein ^a^	Selection strategy ^b^	Library type ^c^	Selected peptide(s) ^d^	Biological activity	Affinity	Potential applications (Biological effects)	Ref.
IL-1R type I	a3	3 and 8	peptides with *C*-terminal motif YWQPYALPL	antagonists	IC_50_ 2-500 nM	therapy of autoimmune and inflammatory disorders (anti-inflammatory effects)	[34]
IL-6Rα	a1	3	LSLITRL	antagonist	IC_50_ >30 μM	cancer therapy (preventing the anti-apoptotic and angiogenic effects of IL-6)	[8]
IL-11Rα	e	3	**C**GRRAGGS**C**	agonist	N/A	cancer therapy (targeted delivery of therapeutic or diagnostic agents to prostate tumors; prevention of chemotherapy-induced thrombocytopenia)	[19,66,73]
FGFR	b	3	KRTGQYKL	antagonist	IC_50_ ~5 nM	cancer therapy (inhibition of angiogenesis and tumor progression)	[68]
EGFR2 (ErbB-2)	a2	3	KCCYSL	N/A	Kd ~30 μM	cancer therapy (targeted delivery of therapeutic or diagnostic agents to tumors)	[79,89]
EGFR	c5	3	N/A	indirect antagonist (decoy receptor)	N/A	cancer therapy (inhibition of tumor cell proliferation)	[82]
VEGFR (KDR)	a1	3	HTMYYHHYQHHL	antagonist	IC_50_ >30 μM	cancer therapy, treatment of diabetic retinopathy (inhibition of angiogenesis and cellular proliferation)	[9]
VEGFR (KDR)	b, d	3	ATWLPPR	antagonist	IC_50_ ~80 μM	cancer therapy, treatment of diabetic retinopathy (inhibition of angiogenesis and cellular proliferation)	[80]
VEGFR (KDR and Flt-1)	c6	8 + 8 and 3 + 3	GERW**C**FDGPRAW-V**C**GWEI, GGNE**C**DIARMWE-WE**C**FERL, RGWVEI**C**AADDY-GR**C**LTEAQ	indirect antagonists (decoy receptors)	IC_50_ ~0.7-7 μM	cancer therapy, treatment of diabetic retinopathy (inhibition of angiogenesis and cellular proliferation)	[85]
VEGFR	c5	3	WHLPFKC, WHKPFRF	indirect antagonists (decoy receptors)	K_d_ ~2.7 μM	cancer therapy, treatment of diabetic retinopathy (inhibition of angiogenesis and cellular proliferation)	[84]
VEGFR1 and NRP1	d	3	**C**PQPRPL**C**	antagonist	N/A	cancer therapy, treatment of diabetic retinopathy (inhibition of angiogenesis and cellular proliferation)	[35,88,90]
IFNAR	a4, b	3	SVQARWEAAFDL-DLY	agonist	IC_50_ ~50 μM	study of the mechanism of IFNAR activation	[65]
IFNAR	d, b	3	SLSPGLP, FSAPVRY	antagonists	N/A	treatment of autoimmune diseases, study of ligand-receptor interactions	[67]
EpoR	a3	8 + 8 and 3 + 3	GGTYS**C**HFGPLT-WV**C**KPQGG	agonist	IC_50_ ~0.2 μM	treatment of anemia, pure red cell aplasia resulting from anti-Epo antibodies (stimulation of erythropoiesis)	[6]
TpoR	a3	8 + 8 (and non-phage libraries)	GG**C**ADGPTLREW-ISF**C**GG	agonist	IC_50_ ~60 nM	treatment of idiopathic thrombocytopenic purpura, thrombocytopenia (stimulation of thrombopoiesis)	[7]
GlyRα1	d	3	YESIRIGVAPSQ **(and others)**	pos./neg. allosteric modulators	N/A	treatment of alcoholism, leads for developing anesthetics (inhibition/enhancement of ethanol activity)	[12]
CCR5	d	3	AFDWTFVPSLIL	antagonist	IC_50_ ~2.6 μM	treatment of multiple sclerosis, rheumatoid arthritis, HCV and HIV infections, prevention of renal allograft rejection (anti-inflammatory effects, prevention of HIV-1 entry to CD4+ cells)	[49]
DR5	a4	3	**C**KVILTHR**C**	antagonist	K_d_ ~272 nM	therapy of neurodegenerative disorders (inhibition of TRAIL-induced apoptosis in neuronal cells)	[37]
DR5	a1	8 + 8 and 3 + 3	QEV**C**MTSCDKLM-K**C**NWMAAM	agonist	IC_50_ ~6 nM	cancer therapy (triggering of apoptosis in tumor cells)	[74]
PMCA4	a1, a2	3	TAWSEVLDLLRR	allosteric inhibitor	K_i_ ~2.3 μM	study of physiological PMCA4 function, study of arterial hypertension mechanisms and retinopathies, development of new class of contraceptives	[38,91,92,93]
B-cell maturation antigen	c5, c6	8 + 8	SS**C**ESPEVDYLE-**C**LY, LQ**C**RYDQLIEEW-R**C**EY **(and others)**	indirect antagonists (decoy receptors)	IC_50_ 0.49-27 μM	cancer therapy (inhibition of APRIL (a proliferation-inducing ligand)-stimulated proliferation)	[83]
αVβ3 integrin, unknown [RD cell line]	d	T7Select 415-1b	**C**QQSNRGDRKR**C**, **C**MGNKRSAKRP**C**	N/A	N/A	cancer therapy (targeted delivery of therapeutic or diagnostic agents to rhabdomiosarcoma)	[86]
unknown [HT-1376 cells from human bladder carcinoma xenografts]	d	T7Select 415-1b	**C**SNRDARR**C**	N/A	N/A	cancer therapy (targeted delivery of therapeutic or diagnostic agents to bladder cancer)	[47]
unknown [NCI-H1299 cell line]	d	3	EHMALTYPFRPP	N/A	N/A	cancer therapy (targeted delivery of therapeutic or diagnostic agents to NSCLC ^e^ cells)	[94]
unknown [HepG2 cell line]	d	3	FLLEPHLMDTSM	N/A	N/A	cancer therapy (targeted delivery of therapeutic or diagnostic agents to hepatocellular carcinoma)	[45]
unknown [MDA-MB-435 cells from breast cancer xenograft]	d, e	T7Select 415-1b	**C**GNKRTRG**C**	N/A	N/A	cancer therapy (targeted delivery of therapeutic or diagnostic agents to tumor lymphatics)	[51]
unknown [MDA-MB-231 cell line]	d	8 + 8	**C**ASPSGALRS**C**	N/A	N/A	cancer therapy (targeted delivery of therapeutic or diagnostic agents to breast cancer)	[46]

**^a^** target protein abbreviations: IL-1R, interleukin-1 receptor; IL-6Rα, α subunit of interleukin-6 receptor; IL-11Rα, α subunit of interleukin-11 receptor; FGFR, fibroblast growth factor receptor; EGFR2, epidermal growth factor receptor 2; VEGFR, vascular endothelial growth factor receptor; KDR, kinase insert domain-containing receptor; Flt-1, *fms*-like tyrosine kinase-1; NRP1, neuropilin-1; IFNAR, interferon-α/β receptor; EpoR, erythropoietin receptor; TpoR, thrombopoietin receptor; GlyRα1, glycine receptor α1; CCR5, C-C chemokine receptor 5; DR5, death receptor 5; PMCA4, plasma membrane Ca^2+^ ATPase 4; **^b^** code according to classification in Figure 1; **^c^** filamentous phage library type according to classification by Petrenko and Smith [2]; **^d^** cysteines forming intramolecular disulfide bridges are depicted in bold; **^e^** NSCLC, non-small cell lung cancer
